# Small Triple-Negative and HER2-Positive Early Breast Cancer: Upfront Surgery or Neoadjuvant Chemotherapy?

**DOI:** 10.3390/cancers18152422

**Published:** 2026-07-28

**Authors:** Gilles Houvenaeghel, Laura Sabiani, Catherine Bouteille, Olivia Quilichini, Didier Cowen, Monique Cohen, Maxime Souquet Bressand

**Affiliations:** 1 Department of Gynecology, Hopital Européen, 6 Rue Désirée Clary, 13003 Marseille, France; o.quilichini@hopital-europeen.fr; 2 Department of Gynecology, Hôpital de la Conception, AP-HM, 147 Bd Baille, 13005 Marseille, France; laurasabiani@hotmail.com; 3 Department of Surgical Oncology, Centre Recherche Cancérologie Marseille, Institution Paoli-Calmettes, 13009 Marseille, France; catherine.bouteille@hotmail.fr (C.B.); cohenm@ipc.unicancer.fr (M.C.); 4 Department of Radiotherapy, Hôpital Européen, Institut Provence Radiotherapie Oncologie Stéréotaxie (IPROS), 6 Rue Désirée Clary, 13003 Marseille, France; d.cowen@hopital-europeen.fr; 5 Department of Medical Oncology, Hopital Européen, 6 Rue Désirée Clary, 13003 Marseille, France; souquet.bressand.maxime@gmail.com

**Keywords:** breast cancer, triple-negative, Her2-positive, neoadjuvant chemotherapy

## Abstract

Breast cancer (BC) can be treated by combinations of surgery, radiotherapy, chemotherapy, endocrine therapy and targeted therapies. High-risk patients, including all patients with axillary involved lymph nodes with triple-negative BC (TNBC) and Her2-positive BC, are treated by neoadjuvant chemotherapy (NAC). For high-risk patients, immunotherapy for TNBC, trastuzumab and pertuzumab then trastuzumab deruxtecan for Her2-positive BC have improved survival. In this narrative review, we will discuss therapy for small triple-negative and HER2-positive early BC without involved or suspicious axillary lymph nodes. Upfront surgery is recommended for cT1a-b cN0 usN0 TNBC and Her2-positive BC with adjuvant therapy for pT1b pN0 BC, discussed case by case for pT1a pN0 Her2-positive BC. No adjuvant chemotherapy is considered for pT1a TNBC. However, lymph vascular invasion can also be contributive on pathologic results. For pT1c pN0 TNBC and Her2-positive BC, adjuvant therapy is recommended. For cT2 or cN+ TNBC and Her2-positive BC, NAC is the treatment recommended with adjuvant therapy after surgery according to pathologic results. For cT1c cN0 usN0 TNBC and Her2-positive BC, upfront surgery is the usual treatment proposed but NAC can be strongly considered according to clinic pathologic characteristics, tumor size 10–15 mm or 16–20 mm, grade, young age (≤35-years) and co-morbidities, particularly for elderly patients. Recent tools such as TIL level determined on core needle biopsy may help when considering NAC or upfront surgery and systemic therapy regimens. Other prognostic tools can contribute to the determined therapeutic strategy and systemic therapy regimens with escalation or de-escalation, such as TNBCDX or HER2DX, possibly in combination with TIL level.

## Simple Summary

Breast cancer (BC) can be treated by combinations of surgery, radiotherapy, chemotherapy, endocrine and targeted therapies. High-risk patients with triple-negative BC (TNBC) and Her2-positive BC are treated by neoadjuvant chemotherapy (NAC). In this narrative review, we discuss therapy for small TNBC and HER2-positive BC without involved or suspicious axillary lymph nodes: upfront surgery is recommended for cT1a-b cN0 usN0 TNBC and Her2-positive BC with adjuvant therapy for pT1b-c pN0 BC, discussed for pT1a pN0. For cT2 or cN+, NAC is recommended with adjuvant therapy after surgery according to pathologic results. For cT1c cN0 usN0 TNBC and Her2-positive BC, upfront surgery is usually proposed but NAC can be strongly considered according to clinic pathologic characteristics. Recent tools such as TNBCDX or HER2DX and TIL may help when considering NAC or upfront surgery and systemic therapy regimens.

## 1. Introduction

Breast cancer (BC) is the most common malignant tumor currently affecting women’s health worldwide [1], the most frequent cancer in French women and the leading cause of cancer-related death in women [2].

BC can be treated by combinations of surgery, radiotherapy, chemotherapy, endocrine therapy and targeted therapies. High-risk patients, including all patients with axillary involved lymph nodes (clinically and or on imagery) with triple-negative BC (TNBC) and Her2-positive BC, are treated by neoadjuvant chemotherapy (NAC). Recently, for high-risk patients, immunotherapy, particularly pembrolizumab in combination with NAC for TNBC [3], trastuzumab and pertuzumab [4,5] then trastuzumab deruxtecan [6] in combination with NAC for Her2-positive BC have improved survival.

In this narrative review, without a comprehensive systematic search, we will discuss therapy for small triple-negative and HER2-positive early BC without involved or suspicious axillary lymph nodes. The literature search was performed using PubMed from 2006 with key words “breast cancer”, “triple negative breast cancer”, “Her2 positive breast cancer”, and “Neo adjuvant chemotherapy”.

## 2. TNBC Rate and Her2-Positive Rate (Table 1)

In a French cohort, among 235,368 BC patients treated between 2010 and 2018 with surgery for all patients [2], BC subtype was determined in 190,980 BC cases: 9.5% TNBC, 6.6% endocrine receptor-positive/Her2-positive (ER+ HER2+) (6.6%), 3.7% ER- HER2+, and 80.2% Luminal BC HER2-negative (2). Chemotherapy was administered in 90,252 patients (38.3%): 72,939 adjuvant chemotherapy (80.8%), 15,627 neoadjuvant chemotherapy (17.3%) and both in 1686 (1.9%). Anti-HER2 target therapy was done in 19,722 BC, 15,298 (77.6%) for adjuvant therapy and 4424 (22.4%) for neoadjuvant therapy followed by adjuvant therapy (19,289 BC patients with trastuzumab only: 97.8%). Endocrine therapy was administered in 70.4% (165,655 BC).

Distribution of BC according to age was significantly different: 5.24% (10.007/190.974) < 40 years, 70.06% (133.800/190.974) 40–69 years and 24.70% (47.167/190.974) ≥ 70 years. Age repartition differed according to BC subtypes: for patients <40 years, 40–69 years and ≥70 years, respectively, TNBC was observed in 23.9% (2390/10.007), 10% (13.372/133.800) and 5.05% (2381/47.167); ER+ HER2+ in 13.7% (1368/10.007), 7.1% (9540/133.800) and 3.5% (1653/47.167); and ER- HER2+ in 6.5% (652/10.007), 4.0% (5364/133.800) and 2.4% (1145/47.167) (2).

In a retrospective multicenter French cohort [7], among 12.572 patients with clinically node-negative invasive BC ≤ 30 mm who did not receive neoadjuvant therapy and underwent sentinel lymph node biopsy between 1999 and 2012, BC subtype rates were 72% (n = 8998) Luminal A BC, 9% (n = 1178) Luminal B HER2-negative BC, 9% TNBC, 6% ER+ HER2+ BC and 4% ER- HER2+ BC.

**Table 1:** Distribution of breast cancer molecular subtypes according to published cohorts.

BC Subtypes Rates	Dumas [2]		Houvenaeghel [7]		SEER Li Q [8]		Reyal [9]	
	Up-Front and NAC		Up-Front SLNB		Up-Front Surgery		Up-Front SLNB	
	Nb	%	Nb	%	Nb	%	Nb	%
ER+ HER2-	153,109	80.2	10,176	81	93,708	79.5	2139	80.6
TNBC	18,149	9.5	1091	9	10,769	9.1	211	7.9
ER+ HER2+	12,561	6.6	766	6	9926	8.4	217	8.2
ER- HER2+	7161	3.7	450	4	3492	3.0	87	3.3

In the SEER database, among 117.895 BC patients treated between 2010 and 2015 by upfront surgery [8], subtype rates were 79.48% Luminal BC, 9.13% TNBC, 8.42% ER+ HER2+ BC and 2.96% ER- HER2+ BC.

In Reyal et al.’s study [9], there was 7.9% TNBC, 8.2% ER+ HER2+ BC and 3.3% ER- HER2+ BC.

In summary, TNBC rate was approximately 9–10%, 6–8% of patients treated by upfront surgery and SLNB, with a greater rate for young patients < 40 years (around 24%); ER+ Her2+ BC rate was approximately 6–7%, 6–8% of patients treated by upfront surgery and SLNB, with a greater rate for young patients < 40 years (around 14%); and ER- Her2+ BC rate was approximately 3–4%, with a greater rate for young patients < 40 years (around 6.5%).

## 3. Small Early Breast Cancer: T1 ≤ 20 mm (Table 2)

In Reyal et al.’s study [9], mainly T1 BC (2333/ 2630), BC subtypes rates were 80.6% ER+ Her2- BC, 7.9% TNBC, 8.2% ER+ HER2+ BC and 3.3% ER- Her2+ BC. For T1 BC, these rates were 81.6%, 7.2%, 8.1% and 3.1% for ER+ Her2-, TNBC, ER+ Her2+ and ER- Her2+ BC. BC subtypes according to T1a-b-c are reported in Table 2. Among 425 T1 TNBC and Her2+ BC, tumor sizes were 7.8% T1a (33 T1a), 21.6% T1b (92 T1b) and 70.6% T1c (300 T1c).

**Table 2:** Distribution of breast cancer molecular subtypes according to T1 tumor size categories.

BC Subtypes Rates	T1a		T1b		T1c		All T1	
	Nb	%	Nb	%	Nb	%	Nb	%
Houvenaeghel [10]	376	11.6	1335	41.1	1536	47.3	3247	
Luminal A	260	69.1	1130	84.6	1183	77	2573	79.2
Luminal B HER2-	11	2.9	44	3.3	107	7.0	162	5.0
Luminal B HER2+	31	8.2	58	4.3	86	5.6	175	5.4
TNBC	45	12	78	5.8	135	8.8	258	7.9
ER- HER2+	29	7.7	25	1.9	25	1.6	79	2.4
Reyal [9]	139	6.0	769	33.3	1401	60.7	2309	
ER+ HER2-	106	76.3	677	88.0	1101	78.6	1884	81.6
TNBC	13	9.35	35	4.55	119	8.5	167	7.2
ER+ HER2+	12	8.6	37	4.8	137	9.8	186	8.1
ER- HER2+	8	5.75	20	2.6	44	3.1	72	3.1

In a French cohort of patients with T1 BC treated with upfront surgery [10], BC subtype rates were (1) for 376 T1a: 69.1% Luminal A, 2.9% Luminal B HER2-, 8.2% (n = 31) Luminal B HER2+, 12% TNBC and 7.7% (n = 29) ER- HER2+ BC; (2) for 1335 T1b: 84.6% Luminal A, 3.3% Luminal B HER2-, 4.3% Luminal B HER2+, 5.8% TNBC and 1.9% ER- HER2+ BC; and (3) for 1536 T1c: 77% Luminal A, 7% Luminal B HER2-, 5.6% Luminal B HER2+, 8.8% TNBC and 1.6% ER- HER2+ BC. Among 512 T1 TNBC and Her2+ BC, tumor sizes were 20.5% T1a (105 T1a), 31.4% T1b (161 T1b) and 48.0% T1c (246 T1c).

For 23.394 TNBC included in a study from the SEER database [11], the repartition according to tumor size was 43.2% (n = 10.011) T1, 42.4% (n = 9930) T2, 8.8% (n = 2060) T3 and 5.95% (n = 1393) T4.

In summary, TNBC, ER+ Her2+ and ER- Her2+ represent approximately 11%, 8% and 7% of T1a; 10%, 9% and 4% of T1b; and 9%, 8% and 2% of T1c, respectively. T1a, T1b and T1c represent approximately 13%, 27% and 60% of TNBC; 12%, 26% and 62% of ER+ Her2+; and 24%, 30% and 46% of ER- Her2+, respectively.

## 4. Axillary Lymph Node Involvement for TNBC and HER2-Positive BC Treated by Upfront Surgery

Sentinel lymph node biopsy is performed for cT1 cN0 usN0 TNBC and HER2-positive BC treated by upfront surgery. The majority of patients are pN0sn and axillary sentinel lymph node involvement can be detected: isolated tumor cells (ITC ≤ 0.2 mm) or micro-metastases (pN1mi sn > 0.2 mm and ≤2 mm) or macro-metastases (pN1 > 2 mm).

Axillary sentinel lymph node involvement rate differed significantly according to BC subtypes [2,7,8,9,11,12,13,14,15,16,17,18,19]: approximatively 19% for Luminal BC (12.6% to 58.4%), 27% for TNBC (9.5% to 50.4%), 23% for ER+ Her2+ BC (13.7% to 62.5%) and 27% for ER- Her2+ BC (13.5% to 60%) (Table 3).

In the French cohort [2], the proportion of node-positive BC were 21.5% for Luminal BC, 23.8% for TNBC, 28.6% (3592/12.561) for ER+ HER2+ BC and 30.1% for ER- HER2+ BC. Moreover, lymph node involvement decrease from age < 30-years to 70–79 years and increase for patients ≥ 80-years: 23.1% (280/1144), 24.4% (2572/10.539), 21.6% (9314/43.206), 18.8% (10.926/58.003), 16.6% (10.650/64,042), 16.5% (6468/39.163) and 20.8% (4014/19.291) for patients < 30-years, 30–39, 40–49, 50–59, 60–69, 70–79 and ≥80-years, respectively.

In the retrospective multicenter French cohort [7], sentinel lymph node involvement rates by macro-metastases were 20.8% (1873/8998) for Luminal A BC, 38.0% (448/1178) for Luminal B HER2- BC, 24.6% for TNBC, 29.0% for ER+ HER2+ BC and 36.9% for ER- HER2+ BC. Sentinel lymph node involvement rates by macro-metastases were 16.3% (1553/9548) for cT0-1 BC and 50% (1057/2115) for cT2 BC. In logistic regression, odds ratios were 2.13 for pT size 11–20 mm and 6.271 for pT size > 20 mm in comparison with pT size < 11 mm.

In the SEER database, among 117.895 BC patients treated by upfront surgery [8], lymph node involvement rates increase significantly with tumor size: 2.0% (28/1372) for T1mic, 2.6% (269/10,229) for T1a, 5.8% (1616/27.926) for T1b, 12.4% (6062/48.802) for T1c, 21.6% (5922/27.419) for T2 and 34.8% (748/2147) for T3 BC. Lymph node involvement rates were 12.6% for Luminal BC, 9.5% for TNBC, 13.7% for ER+ HER2+ BC and 13.5% for ER- HER2+ BC.

In Reyal et al.’s study [9], sentinel lymph node involvement rates were 34.7% for ER+ HER2- BC, 20.4% for TNBC, 31.6% for ER+ HER2+ BC and 41.5% ER- HER2+ BC.

For TNBC in a study using the SEER database, reported by Qiu et al. [11], 64.7% (15.011/23.394) were pN0, 25.06% (5863/23.394) were pN1, 6.02% (1409/23.394) were pN2 and 4.7% (1111/23.394) were pN3.

In the French cohort of patients with T1 BC treated with upfront surgery [10], axillary lymph node status were (1) for 699 T1a: 90% (n = 629) pN0(i-), 2% (n = 14) pN0(i+), 5.1% (n = 36) pN1mi, 2% (n = 14) 1pN1 and 0.9% (n = 6) > 1pN1; (2) for 2154 T1b: 83.7% (n = 1802) pN0(i-), 2.9% (n = 62) pN0(i+), 8.7% (n = 188) pN1mi, 2.8% (n = 60) 1pN1 and 1.9% (n = 42) > 1pN1; and (3) for 2398 T1c: 73.7% (n = 1767) pN0(i-), 4% (n = 96) pN0(i+), 11.6% (n = 279) pN1mi, 5.1% (n = 123) 1pN1 and 5.5% (n = 133) > 1pN1.

In the French cohort of 1771 patients with HER2-positive BC treated with upfront surgery between January 1991 and December 2013 [20], axillary lymph node status were 1047 pN0 (59.1%), 60 pN0(i+) (3.4%), 118 pN1mi (6.7%), and 546 pN1 with macro-metastases (30.8%). Pathologic tumor size was less than 20 mm for 1082 patients (61.1%).

In summary, axillary lymph node involvement rates were approximatively 27% for TNBC, 23% for ER+ Her2+ BC and 27% for ER- Her2+ BC.

In the SERC trial [21] with randomization of completion axillary lymph node dissection in case of sentinel lymph node involvement, pN0(i+), pN1mi and pN1 sentinel nodes were present in 5.91%, 28.12% and 65.97%, respectively. For 1354 ER+/HER2-, 135 ER+/HER2+, 41 ER-/HER2+ and 97 TNBC, isolated tumor cells rates were 5.5% (n = 75), 6.67% (n = 9), 7.3% (n = 3) and 8.2% (n = 8); micro-metastases rates were 27.5% (n = 373), 27.4% (n = 37), 24.4% (n = 10) and 29.9% (n = 29); and macro-metastases rates were 66.9% (n = 906), 65.9% (n = 89), 68.3% (n = 28) and 61.8% (n = 60), respectively.

## 5. Axillary Lymph Node Involvement for Small TNBC and HER2-Positive BC

In the French cohort of patients with TNBC treated with upfront surgery [22], pT sizes were 6.2% (n = 76) 0–5 mm, 15.4% (n = 190) 5.1–10 mm, 44.6% (n = 549) 10.1–20 mm, 19.8% (n = 243) 20.1–30 mm and 14% (n = 172) > 30 mm. Among these 1232 TNBC patients, axillary lymph node status were 77.2% (n = 951) pN0, 2.8% (n = 35) pN0(I+), 4.5% (n = 55) pN1mi and 15.5% (n = 191) pN1a. According to pT size, pN1 rates were 6.9% for pT size 0–10 mm, 20.1% for pT size 11–20 mm and 35.7% pT size > 20 mm (Table 4).

In Reyal et al.’s study [9], sentinel lymph node involvement rates were (1) for 199 TNBC: 0% for pT size 0–5 mm, 11.4% for pT 5.1–10 mm, 16.8% for pT 10.1–20 mm and 25% for pT 20.1–30 mm; (2) for 82 ER- HER2+ BC: 37.5% for pT size 0–5 mm, 20% for pT 5.1–10 mm, 40.9% for pT 10.1–20 mm and 90% for pT 20.1–30 mm; (3) for 208 ER+ HER2+ BC: 8.3% for pT size 0–5 mm, 24.3% for pT 5.1–10 mm, 29.2% for pT 10.1–20 mm and 63.6% for pT 20.1–30 mm; and (4) for 2044 ER+ HER2- BC: 6.6% for pT size 0–5 mm, 20.4% for pT 5.1–10 mm, 34.1% (376/1101) for pT 10.1–20 mm and 59.4% for pT 20.1–30 mm.

## 6. Adjuvant Chemotherapy Indication for Small BC Treated by Upfront Surgery

In the French cohort of patients with T1 BC treated with upfront surgery [10], the following adjuvant chemotherapy was delivered: (1) whatever the tumor subtype, in 14% (81/708) for T1a BC, in 17% (362/2207) for T1b BC and 34% (810/2508) for T1c BC; (2) for patients with ER+, in 9.4% (42/448) of T1a BC, in 14.3% (271/1898) of T1b and 29.5% (639/2166) of T1c; (3) for patients with ER-negative or unknown ER status, in 31.2% (39/125) of T1a, 52% (89/171) of T1b and 78.1% (168/215) of T1c BC.

For TNBC and HER2-positive BC, adjuvant chemotherapy is indicated for patients with pT1b-c pN0 sn but not considered for pT1a pN0 sn. For TNBC and HER2-positive BC with sentinel node ITC or micro-metastases, adjuvant chemotherapy is indicated whatever the pathologic tumor size.

ITC or micro-metastases and lymphovascular invasion (LVI) had been individualized as negative prognostic factors for TNBC [22] and consequently adjuvant chemotherapy is usually recommended for patients with ≥1 sentinel node ITC or micro-metastases, whatever the tumor size. Adjuvant chemotherapy is also recommended for patients with ≥1 sentinel node macro-metastases.

For HER2-positive BC [20], adjuvant chemotherapy was given to 67.0% of pN0 patients (702/1047), 81.7% of pN0(i+) cases (49/60), 93.2% of pN1mi (110/118) and 93.8% of pN1 with macro-metastases (512/546) (*p* < 0.0001). In a binary logistic regression, receipt of adjuvant chemotherapy was significantly associated with tumor size pT ≥ 20 mm (OR 2.476, *p* < 0.0001), grade 2 (OR 3.288, *p* < 0.0001) and grade 3 (OR 10.040, *p* < 0.0001), lymphovascular invasion (LVI) (OR 2.066, *p* < 0.0001), ER-positivity (OR 0.739, *p* = 0.050), and increasing age categories compared with ≤40 years: >40–50 (OR 0.498, *p* = 0.019), >50–74.9 (OR 0.327, *p* < 0.0001) and ≥75 (OR 0.044, *p* < 0.0001). Compared with pN0, pN1mi (OR 6.727, *p* < 0.0001) and pN1 with macro-metastases (OR 4.657, *p* < 0.0001) were also linked to higher odds of adjuvant chemotherapy, whereas pN0(i+) showed no significant association (OR 1.582, *p* = 0.219).

Macro-metastatic pN1 status was the only nodal variable significantly associated with reduced survival outcomes: OS (HR 1.583, *p* = 0.048), DFS (HR 1.737, *p* = 0.001), RFS (HR 2.044, *p* < 0.0001), and MFS (HR 1.824, *p* = 0.001). Additionally, LVI, pT ≥ 20 mm, ER-negative status, omission of adjuvant chemotherapy, and age ≥ 75 years were all significantly associated with worse OS and DFS.

For pT1a–b tumors with pN0 or with pN0(i+)/pN1mi [20], adjuvant chemotherapy was significantly associated with SBR grade, ER status, age group, LVI and pN status. In binary logistic regression, receipt of adjuvant chemotherapy was significantly linked to SBR grade, ER status, age category, LVI, and pT1b (OR 3.189, *p* < 0.0001). In a multivariate model adjusted for adjuvant chemotherapy, ER status, age group, LVI, pT and pN (pN0(i+) or pN1mi), presence of pN0(i+) or pN1mi was associated with reduced RFS (HR 2.548, *p* = 0.037), while patients who received adjuvant chemotherapy had improved RFS (HR 0.288, *p* = 0.003). Adjuvant chemotherapy rates varied by pT and LVI: 36.0% for pT1a without LVI (45/125), 55.6% for pT1a with LVI (5/9), 61.2% for pT1b without LVI (131/214), and 92.7% for pT1b with LVI (38/41) (*p* < 0.0001). In multivariate analysis, RFS was worse for pT1b tumors without LVI (HR 2.893, 95% CI 1.09–7.67, *p* = 0.033), for patients who did not receive adjuvant chemotherapy (HR 3.758, 95% CI 1.41–9.99, *p* = 0.008), for ER-negative disease (HR 2.702, 95% CI 1.10–6.63, *p* = 0.030), and for patients with pN0(i+) or pN1mi (HR 2.780, 95% CI 1.10–7.04, *p* = 0.031). After adjustment for LVI, ER status, age group, pN and pT with or without adjuvant chemotherapy, RFS remained significantly decreased for pT1b tumors without adjuvant chemotherapy (HR 2.365, 95% CI 1.04–5.36, *p* = 0.039) and for pN0(i+)/pN1mi (HR 2.518, 95% CI 1.03–6.14, *p* = 0.042). Among patients with pT1b disease, only those with pN0 who received adjuvant chemotherapy showed improved RFS compared with pN0 patients who did not receive chemotherapy (HR 0.140, *p* = 0.002).

In a systematic review and meta-analysis [23] including 6927 patients from 12 studies, the addition of trastuzumab and chemotherapy was associated with significantly better DFS and OS in HER2-positive T1abN0 cases compared with no treatment. However, these tumors generally had a good prognosis even without adjuvant therapy, and subgroup pooling was not feasible. A later meta-analysis [24] of pT1a N0 M0 HER2-positive BC showed a non-significant survival advantage for the treated group relative to untreated patients.

A systematic review and meta-analysis reported by Hassing et al. in 2026 [25], in patients with T1a-bN0 TNBC including 15,933 patients, overall survival was significantly improved with chemotherapy (HR 0.63). However, the overall survival benefit was mostly driven by patients with T1b N0 TNBC (HR 0.51, 95% CI 0.34–0.76) with no overall survival benefit in patients with T1a N0 tumors (HR 1.24). Five-year distant recurrence-free survival was 95.93% with chemotherapy versus 91.95% without treatment for T1a-b N0 TNBC. Similar results were reported in a recent meta-analysis [26] of 12 cohort studies including 48,371 patients T1a-b-c N0 TNBC with significantly higher overall survival in T1b N0 (HR 0.73), but not in T1a N0 (HR 0.88).

Several international guidelines recommend no adjuvant chemotherapy for T1a N0 TNBC, except for high-risk criteria as young age (<35 years) or high grade. However, the majority of TNBC are high grade (58%) [27]. For T1b N0 TNBC, adjuvant chemotherapy is recommended [28,29,30,31], particularly for T1b N0 M0 grade 3 TNBC (HR 0.08; *p* = 0.03) [27].

International guidelines [32,33,34,35] for Her2-positive BC recommend adjuvant chemotherapy after upfront surgery, including taxane and trastuzumab for pT1b N0, and no therapy or taxane and trastuzumab discussed case by case for pT1a N0. For cT1c N0, the standard is upfront surgery followed by adjuvant treatment, but consideration can be given to NAC followed by surgery and adjuvant treatment. For ≥cT2 or cN+, the standard is NAC with trastuzumab + pertuzumab + chemotherapy. For cN0 usN0 patients treated with upfront surgery but with nodal involvement in the pathological specimen, the standard is chemotherapy + trastuzumab with consideration given to the addition of pertuzumab. For patients with pCR after NAC, the standard is trastuzumab for a total of 1 year. For patients with residual invasive disease in breast and/or axillary lymph node after NAC, the standard is trastuzumab emtansine (T-DM1) [36].

## 7. Chemotherapy According to BC Subtypes

In the French cohort reported by Dumas et al. [2], chemotherapy was administered in 34.3% (52.518/153.109) of luminal BC, 100% (18,149) of TNBC, 98.9% (12.424/12.561) of ER+ HER2+ BC and 100% (7161) of ER- HER2+ BC.

Adjuvant chemotherapy and NAC, respectively, was administered in 85.5% (44.899/52.518) and 14.5% (7619/52.518) of Luminal BC, 72.6% (13.174/18.149) and 27.4% (4975/18.149) of TNBC, 78.0% (9690/12.424) and 22.0% (2734/12.424) of ER+ HER2+ BC, and 72.3% (5176/7161) and 27.7% (1985/7161) of ER- HER2+ BC.

For TNBC, in a study using the SEER database [11], chemotherapy (adjuvant or NAC) was administered in 80.4% (18.801/23.394), and there was no chemotherapy or undetermined treatment in 19.6% (n = 4593).

In study reported in ESMO open [37] for patients treated by upfront surgery, adjuvant chemotherapy rates were 36.2% (5308/14,655) for ER+ BC and 62.6% (1670/2667) for ER- BC.

## 8. Pathologic Complete Response (pCR) After NAC and Tumor Size

No residual tumor on the breast specimen and on axillary lymph nodes are considered as pCR. Residual cell burden (RCB) > 1 with invasive breast tumor or lymph node tumor cells are considered as no pCR. pCR rates are not significantly associated with initial tumor size, determined by clinical exam, breast imagery with mammography, ultrasonography, and sometimes also with magnetic resonance imagery (MRI) or PET-CT when is it performed.

pCR rates were significantly associated in multivariate analysis with tumor subtypes, clinical N stage but not with tumor size (cT1 versus cT > 1) [38], and pCR rates were not significantly associated with HER2-0 or HER2-low (HER2 = 1 or 2) [39].

In a study reported by Houvenaeghel et al. [38], pCR rates for breast tumors (ypT0-is) treated between January 2005 and May 2021 were 14.2% (56/393) for Luminal A BC, 28.8% (57/198) for Luminal B HER2-, 49.0% (148/302) for Luminal B HER2+, 67.7% (149/220) for ER- HER2+ and 49.1% (252/513) for TNBC without significant difference according to tumor size: 45.4% (109/240) for cT0-1, 41.7% (390/935) for cT2, 37.1% (130/350) for cT3 and 33.6% (35/104) for cT4. Moreover, no pCR had a significant negative survival impact on OS, DFS and MFS (HR: 4.972, 4.717, and 4.673, respectively) for TNBC and on OS (HR: 11.706) for HER2+ ER+ or ER- BC, but cT size was not significant (cT1 vs. >cT1) for TNBC, HER2+ [40].

## 9. Contribution to Detection of Occult Lymph Node Metastases

Occult lymph node metastases can be detected on sentinel lymph nodes, ITC or micro-metastases by serial section analysis [41] and immunochemistry when serial sections do not detect tumor cells. Considering a negative impact factor of occult metastases for small TNBC [22] and HER2-positive BC after upfront surgery, their detection appears useful for indication of adjuvant chemotherapy. For patients treated by NAC, detection of occult lymph node metastases (ypN0(i+) and ypN1mi) also appears useful for indication of adjuvant chemotherapy after surgery.

In the SERC trial [21] for patients with SN micro metastases, 29 tumors were triple-negative (6.46%), 10 ER- Her2+ (2.23%), and 37 ER+ Her2+ (8.24%), and in the G3S cohort for SN micro-metastasis patients, 64 tumors were TNBC (5.40%), 28 ER- Her2+ (2.30%), and 67 ER+ Her2+ (5.60%). In the SENOMIC study, 49 tumors were ER- (8.9%) and 61 Her2+ (11.1%) tumors were reported, and in the NKBC cohort, 96 (9.0%) were ER- and 140 (13.1%) were Her2+.

However, completion axillary lymph node dissection is not recommended for pN0(i+) sentinel node or for pN1mi sentinel node after upfront surgery, even if the evidence level is low for patients treated by mastectomy without indication of post-mastectomy radiotherapy. Indeed, the number of patients treated by mastectomy with ITC or micro-metastases sentinel nodes included in trials is very low: 86 in the IBCSG 23-01 trial [42,43], 18 in AATRM [44], 82 in the SERC trial [21] for randomized trials, 204 in the cohort SENOMIC without completion ALND [45], 274 in the G3S cohort [46], and 420 in the NKBC cohort [45]. Tumor subtypes were not recorded in the IBCSG and AATRM trials.

For patients treated by NAC, completion axillary lymph node dissection can be allowed, since the results of the OPBC-07/microNAC trial showed no significant survival difference between those who underwent cALND and those who did not [47]. However, a significant greater axillary recurrence rate was reported for TNBC without cALND in comparison to with cALND [48].

## 10. Adjuvant Chemotherapy After NAC for Small TNBC and HER2-Positive BC

### 10.1. Patients Without Axillary Lymph Node Involvement (ypN0)

For patients with TNBC, adjuvant systemic treatment includes chemotherapy with pembrolizumab immunotherapy for high-risk patients with residual tumor burden.

For patients with HER2-positive BC, two targeted therapies have been approved for early BC: trastuzumab and pertuzumab depending on the country. Combinations of target therapy and adjuvant systemic treatments were (1) anthracycline and then a combination of docetaxel and target therapy; (2) anthracycline and then a combination of paclitaxel and target therapy; (3) combination of docetaxel and target therapy; (4) combination of paclitaxel and target therapy, as described by Tolaney et al. [49]; and (5) combination of endocrine therapy and target therapy, without chemotherapy. The combination of trastuzumab and pertuzumab is approved for high-risk BC, ypN1-2. For patients with HER2-positive BC without residual tumor burden and small tumors (cT1a-b-c), adjuvant trastuzumab only is proposed.

### 10.2. Patients with Axillary Lymph Node Involvement (cN+)

For patients with TNBC and ypN0(i+) sn after NAC, completion ALND can be discussed, and adjuvant chemotherapy should be proposed. However, there is few data for this situation. For TNBC and ypN1mi sn, completion ALND is actually proposed, taking into account the results of the OPBC-07/microNAC prospective trial, which had a significantly greater axillary recurrence rate without cALND in comparison to with cALND [48]. Adjuvant chemotherapy is proposed due to the presence of a residual nodal tumor burden. For TNBC and ypN1a sn high-risk BC, completion ALND and adjuvant chemotherapy are recommended.

For patients with axillary lymph node involvement (cN+) HER2-positive BC with ypN0(i+) sn or ypN1mi sn, completion ALND can be omitted since the results of the OPBC-07/microNAC trial showed no significant survival difference between whether completion ALND was performed or not [47]. Adjuvant therapy is recommended due to the presence of a residual nodal tumor burden. For HER2-positive BC and pN1a high-risk BC, completion ALND and adjuvant therapy including trastuzumab emtansine are recommended.

Patients with residual tumor burden (no pCR) benefit from intensification with adjuvant therapy, and conversely, patients without residual tumor burden (pCR) may be able to receive less intensive chemotherapy.

However, adjuvant multi-agent chemotherapy has not been tested in randomized trials in this setting.

## 11. Indication of NAC for TNBC and HER2-Positive BC cN0 usN0 ≥ 10 mm

Performing NAC thus allows for the selection of non-pCR patients with a more unfavorable prognosis compared to patients with pCR, who are treated after surgery by adjuvant therapy in order to improve survival results. For patients with residual breast tumor or residual lymph node tumor after NAC, improvement of survival has been highlighted with adjuvant therapy by several randomized trials. Moreover, patients with upfront surgery can be under-evaluated for axillary lymph node status with detection of small sentinel lymph node involvement (pN0(i+) or pN1mi or pN1a) in approximatively 14 to 34% of cases for TNBC and Her2-positive BC.

Identification of prognostic biomarkers may help risk assessment for BC recurrence after upfront surgery and predict pCR rate after NAC, such as for tumor-infiltrating lymphocytes (TILs). High TIL rates correspond to a better prognosis compared to low rates: in a cohort of 1041 TNBC patients [50], TIL levels ≥ 30% were significantly associated with better breast cancer-specific survival compared with TIL levels < 30% (HR 0.45). For example, patients with T1bN0 TNBC and TIL levels ≥ 50% had improved prognosis with both 5-year OS and DRFS of 97% [51].

In the review by Schlam et al. in 2025 [52], higher TIL levels were associated with better recurrence-free survival in Her2-positive BC after NAC with higher pCR rate and for upfront surgery with adjuvant therapy. Due to their prognostic value, TILs should be included in pathology reports, but use in clinical practice needs further validation to determine the optimal TIL threshold and how to incorporate the criteria with other clinics’ pathologic characteristics.

In the meta-analysis by Xia et al. [53], pCR rate was higher in patients with high TIL levels than in patients with low TIL levels at different expression thresholds (10% [RR = 1.99], 20-/30% [RR = 1.57], 50–60% [RR = 1.91]). However, Dieci et al. [54] reported a randomized trial that showed an independent effect of TILs in terms of overall survival for Her2-positive BC patients treated with adjuvant chemotherapy and anti-ERBB2 therapy with a 20% TIL threshold.

Among other prognostic tools investigated, a prognostic score, HER2DX—including tumor size, nodal staging, 27 genes, and four gene expression signatures—was validated. This HER2DX score has strong prognostic value, even for small tumors < 3 cm, which helps distinguish low- and high-risk BC and guide systemic treatment type and indication [55,56]. In the meta-analysis reported by Villacampa et al. [57] including 11 studies and 2518 patients, the HER2DX low-risk group had a 6-year event-free survival rate of 93.6% compared with 82.9% for the HER2DX high-risk group (HR 2.72, *p* < 0.0001). This association was consistent across subgroups, regardless of tumor and nodal stage, pCR, and endocrine receptor status. The authors concluded that these results support the use of the HER2DX score in tailoring treatment intensity and guiding clinical decision-making.

A TNBC-DX test that combines the 10-gene Core Immune Gene module, a four-gene tumor cell proliferation signature, tumor size, and nodal stage was developed [58]. The pCR rates for TNBC-DX categories were 56.3% for pCR-high, 53.6% for pCR-medium, and 22.5% for pCR-low.

In a recent study by Stecklein et al. [59], both stromal TILs and the TNBC-DX score independently predicted pCR in patients treated with docetaxel–carboplatin plus pembrolizumab. Patients with stromal TILs ≥ 30% and a TNBC-DX pCR-high genomic score had a pCR rate of 91.3%. A combined classification using TILs and TNBC-DX identified roughly 40% of patients who achieved a pCR rate above 85%.

TIL analyses are easily performed and accessible with acceptable reproducibility [52,53,54] and low cost with routine clinical application. However, high-cost HER2DX and TNBC-DX assays cannot currently be used in routine clinical practice.

## 12. Conclusions

**Figure 1:**
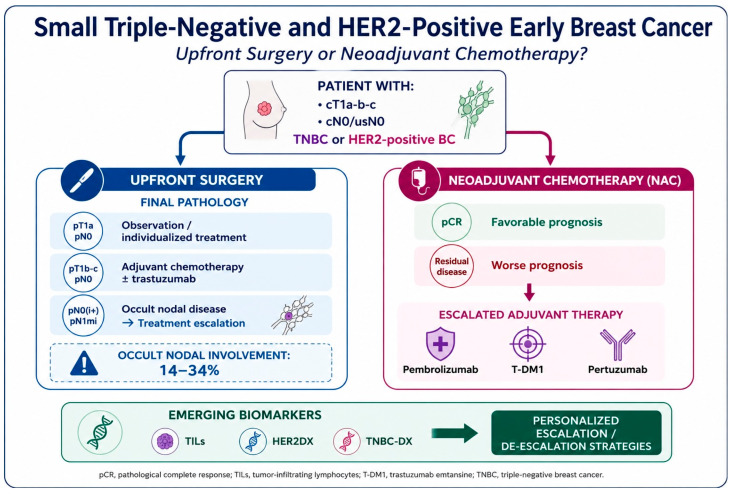
Treatment of small triple-negative and HER2-positive early breast cancer.

In conclusion, upfront surgery is recommended for cT1a-b cN0 usN0 TNBC and Her2-positive BC with adjuvant therapy for pT1b pN0 BC, discussed case by case for pT1a pN0 Her2-positive BC. No adjuvant chemotherapy is considered for pT1a TNBC. However, lymph vascular invasion can also contribute to pathologic results. For pT1c pN0 TNBC and Her2-positive BC, adjuvant therapy is recommended (Figure 1). For cT2 or cN+ TNBC and Her2-positive BC, NAC is the treatment recommended, with adjuvant therapy after surgery according to pathologic results. For cT1c cN0 usN0 TNBC and Her2-positive BC, upfront surgery is the usual treatment proposed but NAC can be strongly considered according to clinic pathologic characteristics, tumor size 10–15 mm or 16–20 mm, grade, young age (≤35-years) and co-morbidities, particularly for elderly patients. Recent tools may help when considering NAC or upfront surgery, and systemic therapy regimens such as TIL level determined on core needle biopsy. Other prognostic tools, such as TNBCDX or HER2DX, possibly in combination with TIL level, can contribute in the determination of therapeutic strategy, NAC or upfront surgery, and systemic therapy regimens with escalation or de-escalation.

## Figures and Tables

**Table 3:** Axillary lymph node involvement according to breast cancer subtype.

LN+ Rate		Luminal	TNBC	ER+ HER2+	ER- HER2+
Dumas [2]	Nb	153,109	18,149	12,561	7161
	Nb LN+	32,893	4313	3592	2152
	%	21.5	23.8	28.6	30.1
Houvenaeghel [7]	Nb	10,176	1091	766	450
	Nb LN+	2321	268	222	166
	%	22.8	24.6	29.0	36.9
Li Q [8]	Nb	93,708	10,769	9926	3492
	Nb LN+	11,792	1023	1358	472
	%	12.6	9.5	13.7	13.5
Reyal [9]	Nb	1264	147	79	53
	Nb LN+	439	30	25	22
	%	34.7	20.4	31.6	41.5
Lu [12]	Nb	60	38	16	15
	Nb LN+	35	11	10	9
	%	58.4	29.0	62.5	60.0
Crabb [13]	Nb	2397	315	210	240
	Nb LN+	1000	116	115	131
	%	41.5	36.7	54.8	68.0
Liu [14]	Nb		477		149
	Nb LN+		240		81
	%		50.4		54.4
Van Calster [15]	Nb	1778	193	168	88
	Nb LN+	639	67	85	32
	%	36.0	34.7	50.6	36.4
Lee [16]	Nb	189	58	54	41
	Nb LN+	46	25	16	14
	%	24.4	43.2	29.6	34.2
Kim [17]	Nb	345	237	61	133
	Nb LN+	174	119	38	72
	%	50.4	50.3	62.3	54.1
Nguyen [18]	Nb	595	89	77	32
	Nb LN+	155	30	28	16
	%	26.0	34	36.0	50.0
Voduc [19]	Nb	2017	556	227	185
	Nb LN+	863	206	125	100
	%	42.8	37.1	55.1	54.1
Qiu [11]	Nb		23,394		
	Nb LN+		8383		
	%		35.8		
Total	Nb	265,638	55,513	24,145	12,039
	Nb LN+	50,357	14,831	5614	3281
	%	18.96	26.72	23.25	27.25

**Table 4:** Axillary lymph node involvement according to tumor size in small TNBC and HER2-positive breast cancer.

LN+ Rates	pT1a			pT1b			pT1C			pT1a-b-c			pT2 ≤ 30 mm		
	Nb	Nb LN+	%	Nb	Nb LN+	%	Nb	Nb LN+	%	Nb	Nb LN+	%	Nb	Nb LN+	%
Reyal [9]															
TNBC	15	0		35	4	11.4	119	20	16.8	169	24	14.2	32	8	25.0
ER+ HER2+	12	1	8.3	37	9	24.3	137	40	29.2	186	50	26.9	22	14	63.6
ER- HER2+	8	3	37.5	20	4	20.0	44	18	40.9	72	25	34.7	10	9	90.0
ER+ HER2-	106	7	6.6	677	138	20.4	1101	376	34.1	1884	521	27.6	160	95	59.4
All subtypes	141	11	7.8	769	155	20.2	1401	454	32.4	2311	620	26.8	224	126	56.3
Houvenaeghel [22]															
TNBC	259	18	6.9	547	110	20.1	414	148	35.7	1220	276	22.6			

## Data Availability

No new data were created or analyzed in this study. Data sharing is not applicable to this article.

## References

[1] Bray F., Laversanne M., Sung H., Ferlay J., Siegel R.L., Soerjomataram I., Jemal A. (2024). Global cancer statistics 2022: GLOBOCAN estimates of incidence and mortality worldwide for 36 cancers in 185 countries. CA Cancer J. Clin..

[2] Dumas E., Laot L., Coussy F., Grandal Rejo B., Daoud E., Laas E., Kassara A., Majdling A., Kabirian R., Jochum F. (2022). The French Early Breast Cancer Cohort (FRESH): A Resource for Breast Cancer Research and Evaluations of Oncology Practices Based on the French National Healthcare System Database (SNDS). Cancers.

[3] Schmid P., Cortes J., Dent R., McArthur H., Pusztai L., Kümmel S., Denkert C., Park Y.H., Hui R., Harbeck N. (2024). Overall Survival with Pembrolizumab in Early-Stage Triple-Negative Breast Cancer. N. Engl. J. Med..

[4] Piccart M., Procter M., Fumagalli D., de Azambuja E., Clark E., Ewer M.S., Restuccia E., Jerusalem G., Dent S., Reaby L. (2021). Adjuvant Pertuzumab and Trastuzumab in Early HER2-Positive Breast Cancer in the APHINITY Trial: 6 Years’ Follow-Up. J. Clin. Oncol..

[5] Loibl S., Jassem J., Sonnenblick A., Parlier D., Winer E., Bergh J., Gelber R.D., Restuccia E., Im Y.H., Huang C.S. (2024). Adjuvant Pertuzumab and Trastuzumab in Early Human Epidermal Growth Factor Receptor 2-Positive Breast Cancer in the APHINITY Trial: Third Interim Overall Survival Analysis with Efficacy Update. J. Clin. Oncol..

[6] Harbeck N., Modi S., Pusztai L., Ohno S., Wu J., Kim S.B., Yoshida A., Fabi A., Cao X., Joseph R. (2026). Neoadjuvant trastuzumab deruxtecan alone or followed by paclitaxel, trastuzumab, and pertuzumab for high-risk HER2-positive early breast cancer (DESTINY-Breast11): A randomised, open-label, multicentre, phase III trial. Ann. Oncol..

[7] Houvenaeghel G., Lambaudie E., Classe J.M., Mazouni C., Giard S., Cohen M., Faure C., Charitansky H., Rouzier R., Daraï E. (2019). Lymph node positivity in different early breast carcinoma phenotypes: A predictive model. BMC Cancer.

[8] Li Q., Xu H., Bao B., Xie Y., Guo S., Gao Z., Chen S., Sun J., Zhu L., Wang J. (2025). Predicting sentinel lymph node metastasis in breast cancer: A study based on the SEER database. Clin. Exp. Med..

[9] Reyal F., Rouzier R., Depont-Hazelzet B., Bollet M.A., Pierga J.Y., Alran S., Salmon R.J., Fourchotte V., Vincent-Salomon A., Sastre-Garau X. (2011). The molecular subtype classification is a determinant of sentinel node positivity in early breast carcinoma. PLoS ONE.

[10] Houvenaeghel G., Goncalves A., Classe J.M., Garbay J.R., Giard S., Charytensky H., Cohen M., Belichard C., Faure C., Uzan S. (2014). Characteristics and clinical outcome of T1 breast cancer: A multicenter retrospective cohort study. Ann. Oncol..

[11] Qiu Y., Chen Y., Shen H., Yan S., Li J., Wu W. (2024). Triple-negative breast cancer survival prediction: Population-based research using the SEER database and an external validation cohort. Front Oncol..

[12] Lu X., Lu X., Wang Z.C., Iglehart J.D., Zhang X., Richardson A.L. (2008). Predicting features of breast cancer with gene expression patterns. Breast Cancer Res. Treat..

[13] Crabb S.J., Cheang M.C.U., Leung S., Immonen T., Nielsen T.O., Huntsman D.D., Bajdik C.D., Chia S.K. (2008). Basal breast cancer molecular subtype predicts for lower incidence of axillary lymph node metastases in primary breast cancer. Clin. Breast Cancer.

[14] Liu H., Fan Q., Zhang Z., Li X., Yu H., Meng F. (2008). Basal-HER2 phenotype shows poorer survival than basal-like phenotype in hormone receptor-negative invasive breast cancers. Hum. Pathol..

[15] Van Calster B., Vanden Bempt I., Drijkoningen M., Pochet N., Cheng J., Van Huffel S., Hendrickx W., Decock J., Huang H.J., Leunen K. (2009). Axillary lymph node status of operable breast cancers by combined steroid receptor and HER-2 status: Triple positive tumours are more likely lymph node positive. Breast Cancer Res. Treat..

[16] Lee J.H., Kim S.H., Suh Y.J., Shim B.Y., Kim H.K. (2010). Predictors of Axillary Lymph Node Metastases (ALNM) in a Korean Population with T1-2 Breast Carcinoma: Triple Negative Breast Cancer has a High Incidence of ALNM Irrespective of the Tumor Size. Cancer Res. Treat..

[17] Kim M., Ro J.Y., Ahn S., Kim H.H., Kim S., Gong G. (2006). Clinicopathologic significance of the basal-like subtype of breast cancer: A comparison with hormone receptor and Her2/neu-overexpressing phenotypes. Hum. Pathol..

[18] Nguyen P.L., Taghian A.G., Katz M.S., Niemierko A., Abi Raad R.F., Boon W.L., Bellon J.R., Wong J.S., Smith B.L., Harris J.R. (2008). Breast cancer subtype approximated by estrogen receptor, progesterone receptor, and HER-2 is associated with local and distant recurrence after breast-conserving therapy. J. Clin. Oncol..

[19] Voduc K.D., Cheang M.C.U., Tyldesley S., Gelmon K., Nielsen T.O., Kennecke H. (2010). Breast cancer subtypes and the risk of local and regional relapse. J. Clin. Oncol..

[20] Houvenaeghel G., Cohen M., Martino M., Reyal F., Classe J.M., Chauvet M.P., Colombo P.E., Heinemann M., Jouve E., Gimbergues P. (2023). Negative Survival Impact of Occult Lymph Node Involvement in Small HER2-Positive Early Breast Cancer Treated by Up-Front Surgery. Cancers.

[21] Houvenaeghel G., Cohen M., Raro P., De Troyer J., Gimbergues P., Tunon de Lara C., Ceccato V., Vaini-Cowen V., Faure-Virelizier C., Marchal F. (2021). Sentinel node involvement with or without completion axillary lymph node dissection: Treatment and pathologic results of randomized SERC trial. npj Breast Cancer.

[22] Houvenaeghel G., Sabatier R., Reyal F., Classe J.M., Giard S., Charitansky H., Rouzier R., Faure C., Garbay J.R., Daraï E. (2016). Axillary lymph node micrometastases decrease triple-negative early breast cancer survival. Br. J. Cancer.

[23] Hassing C.M.S., Nielsen D.L., Knoop A.S., Tvedskov T.H.F., Kroman N., Laenkholm A.V., Juhl C.B., Kümler I. (2023). Adjuvant treatment with trastuzumab of patients with HER2-positive, T1a-bN0M0 breast tumors: A systematic review and meta-analysis. Crit. Rev. Oncol. Hematol..

[24] Ibrahim E.M., Refae A.A., Bayer A.M., Abdullah N., Al-Foheidi M.E. (2025). Adjuvant Therapy Benefits for Patients with Human Epidermal Growth Factor Receptor 2-Positive T1aN0M0 Breast Cancer: A Systematic Review and Meta-Analysis. World J. Oncol..

[25] Hassing C.M.S., Juhl C.B., Tvedskov T.H.F., Nielsen D.L., Knoop A.S., Kroman N.T., Lænkholm A.V., Kümler I. (2026). Benefit of adjuvant chemotherapy in patients with T1a-bN0, triple-negative breast cancer—A systematic review and meta-analysis. Crit. Rev. Oncol. Hematol..

[26] de Moraes F.C.A., Wagner P.H.d.S., da Silva A.B.N., Matheus G.T.F.U., Magalhaes M.C.F., Burbano R.M.R. (2025). Adjuvant Chemotherapy in Early Triple-Negative Breast Cancer (T1a-c N0M0): A Systematic Review and Meta-Analysis. Clin. Breast Cancer.

[27] Lo C., Chang D.-Y., Lu Y.-S., Wang M.-Y., Tsai L.-W., Huang C.-S., Tang C.-H., Lin C.-H. (2025). Benefit of adjuvant chemotherapy for T1cN0M0 and selected T1bN0M0 triple-negative breast cancer: A nationwide cancer registry-based study Open Access. Oncologist.

[28] Ditsch N., Gnant M., Thomssen C., Harbeck N. (2025). St. Gallen/Vienna 2025 Summary of Key Messages on Therapy in Early Breast Cancer from the 2025 St. Gallen International Breast Cancer Conference. Breast Care.

[29] Loibl S., Andre F., Bachelot T., Barrios C.H., Bergh J., Burstein H.J., Cardoso M.J., Carey L.A., Dawood S., Del Mastro L. (2024). Early breast cancer: ESMO Clinical Practice Guideline for diagnosis, treatment and follow-up. Ann. Oncol..

[30] (2025). NCCN Clinical Practice Guidelines in Oncology (NCCN Guidelines) Version 5. [WWW Document]. https://www.nccn.org/professionals/physician_gls/pdf/breast.pdf.

[31] Curigliano G., Burstein H.J., Gnant M., Loibl S., Cameron D., Regan M.M., Denkert C., Poortmans P., Weber W.P., Thürlimann B. (2023). St Gallen Consensus Conference Panelists 2023. Understanding breast cancer complexity to improve patient outcomes: The St Gallen International Consensus Conference for the Primary Therapy of Individuals with Early Breast Cancer 2023. Ann. Oncol..

[32] Burstein H.J., Curigliano G., Thürlimann B., Weber W.P., Poortmans P., Regan M.M., Senn H.J., Winer E.P., Gnant M. (2021). Panelists of the St Gallen Consensus Conference. Customizing local and systemic therapies for women with early breast cancer: The St. Gallen International Consensus Guidelines for treatment of early breast cancer. Ann. Oncol..

[33] Denduluri N., Somerfield M.R., Chavez-MacGregor M., Comander A.H., Dayao Z., Eisen A., Freedman R.A., Gopalakrishnan R., Graff S.L., Hassett M.J. (2021). Selection of optimal adjuvant chemotherapy and targeted therapy for early breast cancer: ASCO Guideline Update. J. Clin. Oncol..

[34] (2021). National Comprehensive Cancer Network Clinical Practice Guidelines Oncology. https://www.nccn.org/guidelines/guidelines-detail?category=1&id=1419.

[35] Cardoso F., Kyriakides S., Ohno S., Penault-Llorca F., Poortmans P., Rubio I.T., Zackrisson S., Senkus E., on behalf of the ESMO Guidelines Committee (2019). Early breast cancer: ESMO Clinical Practice Guidelines for diagnosis, treatment and follow-up. Ann. Oncol..

[36] Manna M., Gelmon K.A., Boileau J.F., Brezden-Masley C., Cao J.Q., Jerzak K.J., Prakash I., Sehdev S., Simmons C., Bouganim N. (2024). Guidance for Canadian Breast Cancer Practice: National Consensus Recommendations for the Systemic Treatment of Patients with HER2+ Breast Cancer in Both the Early and Metastatic Setting. Curr. Oncol..

[37] Houvenaeghel G., Cohen M., Classe J.M., Reyal F., Mazouni C., Chopin N., Martinez A., Daraï E., Coutant C., Colombo P.E. (2021). Lymphovascular invasion has a significant prognostic impact in patients with early breast cancer, results from a large, national, multicenter, retrospective cohort study. ESMO Open.

[38] Houvenaeghel G., de Nonneville A., Cohen M., Viret F., Rua S., Sabiani L., Buttarelli M., Charaffe E., Monneur A., Jalaguier-Coudray A. (2022). Neoadjuvant Chemotherapy for Breast Cancer: Evolution of Clinical Practice in a French Cancer Center Over 16 Years and Pathologic Response Rates According to Tumor Subtypes and Clinical Tumor Size: Retrospective Cohort Study. J. Surg. Res..

[39] de Nonneville A., Houvenaeghel G., Cohen M., Sabiani L., Bannier M., Viret F., Gonçalves A., Bertucci F. (2022). Pathological complete response rate and disease-free survival after neoadjuvant chemotherapy in patients with HER2-low and HER2-0 breast cancers. Eur. J. Cancer.

[40] Houvenaeghel G., de Nonneville A., Cohen M., Sabiani L., Buttarelli M., Charaffe E., Jalaguier A., Bannier M., Tallet A., Viret F. (2024). Neoadjuvant chemotherapy for breast cancer: Pathologic response rates but not tumor size, has an independent prognostic impact on survival. Cancer Med..

[41] Houvenaeghel G., Nos C., Mignotte H., Classe J.M., Giard S., Rouanet P., Lorca F.P., Jacquemier J., Bardou V.J., Groupe des Chirurgiens de la Federation des Centres de Lutte Contre le Cancer (2006). Micrometastases in sentinel lymph node in a multicentric study: Predictive factors of nonsentinel lymph node involvement--Groupe des Chirurgiens de la Federation des Centres de Lutte Contre le Cancer. J. Clin. Oncol..

[42] Galimberti V., Cole B.F., Zurrida S., Viale G., Luini A., Veronesi P., Baratella P., Chifu C., Sargenti M., Intra M. (2013). Axillary dissection versus no axillary dissection in patients with sentinel-node micrometastases (IBCSG 23-01): A phase 3 randomised controlled trial. Lancet Oncol..

[43] Galimberti V., Cole B.F., Viale G., Veronesi P., Vicini E., Intra M., Mazzarol G., Massarut S., Zgajnar J., Taffurelli M. (2018). Axillary dissection versus no axillary dissection in patients with breast cancer and sentinel-node micrometastases (IBCSG 23-01): 10-year follow-up of a randomised, controlled phase 3 trial. Lancet Oncol..

[44] Solá M., Alberro J.A., Fraile M., Santesteban P., Ramos M., Fabregas R., Moral A., Ballester B., Vidal S. (2013). Complete axillary lymph node dissection versus clinical follow-up in breast cancer patients with sentinel node micrometastasis: Final results from the multicenter clinical trial AATRM 048/13/2000. Ann. Surg. Oncol..

[45] Andersson Y., Bergkvist L., Rydén L., Celebioglu F., Falck A.K., Norenstedt S., Ruderfors Malterling R., Vikhe Patil E., Frisell J., de Boniface J. (2025). Omitting completion axillary lymph node dissection in breast cancer patients with sentinel lymph node micrometastases undergoing mastectomy: Results from the prospective SENOMIC trial. Br. J. Surg..

[46] Houvenaeghel G., El Hajj H., Barrou J., Cohen M., Raro P., De Troyer J., Gimbergues P., Tunon de Lara C., Ceccato V., Vaini-Cowen V. (2020). External Validation of the SERC Trial Population: Comparison with the Multicenter French Cohort, the Swedish and SENOMIC Trial Populations for Breast Cancer Patients with Sentinel Node Micro-Metastasis. Cancers.

[47] Montagna G., Alvarado M., Myers S., Mrdutt M.M., Sun S.X., Sevilimedu V., Barrio A.V., van den Bruele A.B., Boughey J.C., Boyle M.K. (2026). Oncological outcomes with and without axillary lymph node dissection in patients with residual micrometastases after neoadjuvant chemotherapy (OPBC-07/microNAC): An international, retrospective cohort study. Lancet Oncol..

[48] Montagna G., Laws A., Ferrucci M., Mrdutt M.M., Sun S.X., Bademler S., Balbaloglu H., Balint-Lahat N., Banys-Paluchowski M., Barrio A.V. (2025). Nodal burden and oncologic outcomes in patients with residual isolated tumor cells after neoadjuvant chemotherapy (ypN0i+): The OPBC-05/ICARO study. J. Clin. Oncol..

[49] Tolaney S.M., Barry W.T., Dang C.T., Yardley D.A., Moy B., Marcom P.K., Albain K.S., Rugo H.S., Ellis M., Shapira I. (2015). Adjuvant Paclitaxel and Trastuzumab for Node-Negative, HER2-Positive Breast Cancer. N. Engl. J. Med..

[50] Geurts V.C.M., Balduzzi S., Steenbruggen T.G., Linn S.C., Siesling S., Badve S.S., DeMichele A., Ignatiadis M., Leon-Ferre R.A., Goetz M.P. (2024). Tumor-Infiltrating Lymphocytes in Patients with Stage I Triple-Negative Breast Cancer Untreated with Chemotherapy. JAMA Oncol..

[51] Leon-Ferre R.A., Jonas S.F., Salgado R., Loi S., De Jong V., Carter J.M., Nielsen T.O., Leung S., Riaz N., Chia S. (2024). Tumor-Infiltrating Lymphocytes in Triple-Negative Breast Cancer. JAMA.

[52] Schlam I., Loi S., Salgado R., Swain S.M. (2025). Tumor-infiltrating lymphocytes in HER2-positive breast cancer: Potential impact and challenges. ESMO Open.

[53] Xia G., Zhang Z., Jiang Q., Wang H., Wang J. (2024). Predictive value of stromal tumor-infiltrating lymphocytes in patients with breast cancer treated with neoadjuvant chemotherapy: A meta-analysis. Medicine.

[54] Dieci M.V., Bisagni G., Bartolini S., Schirone A., Cavanna L., Musolino A., Giotta F., Rimanti A., Garrone O., Bertone E. (2025). Tumor-Infiltrating Lymphocytes and Survival Outcomes in Early ERBB2-Positive Breast Cancer: 10-Year Analysis of the ShortHER Randomized Clinical Trial. JAMA Oncol..

[55] Prat A., Guarneri V., Pascual T., Brasó-Maristany F., Sanfeliu E., Pare L., Schettinia F., Martíneza D., Jaresg P., ∙Griguolo G. (2022). Development and validation of the new HER2DX assay for predicting pathological response and survival outcome in early-stage HER2-positive breast cancer. eBioMedicine.

[56] Fernandez-Martinez A., Rediti M., Tang G., Pascual T., Hoadley K.A., Venet D., Rashid N.U., Spears P.A., Islam M.N., El-Abed S. (2024). Tumor intrinsic subtypes and gene expression signatures in early-stage ERBB2/HER2-positive breast cancer: A pooled analysis of CALGB 40601, NeoALTTO, and NSABP B-41 trials. JAMA Oncol..

[57] Villacampa G., Pascual T., Tarantino P., Cortés J., Perez-García J., Llombart-Cussac A., Conte P., Mancino M., Guarneri V., Dieci M.V. (2025). HER2DX and survival outcomes in early-stage HER2-positive breast cancer: An individual patient-level meta-analysis. Lancet Oncol..

[58] Martín M., Stecklein S.R., Gluz O., Villacampa G., Monte-Millán M., Nitz U., Cobo S., Christgen M., Brasó-Maristany F., Álvarez E.L. (2025). TNBC-DX genomic test in early-stage triple-negative breast cancer treated with neoadjuvant taxane-based therapy. Ann. Oncol..

[59] Stecklein S.R., Martín M., Villacampa G., Del Monte-Millan M., Yoder R., Pathak H., Cobo S., Brasó-Maristany F., Álvarez E.L., Echavarría I. (2026). Stromal tumor infiltrating lymphocytes and TNBC-DX provide complementary prognostic information in triple-negative breast cancer. J. Natl. Cancer Inst..

